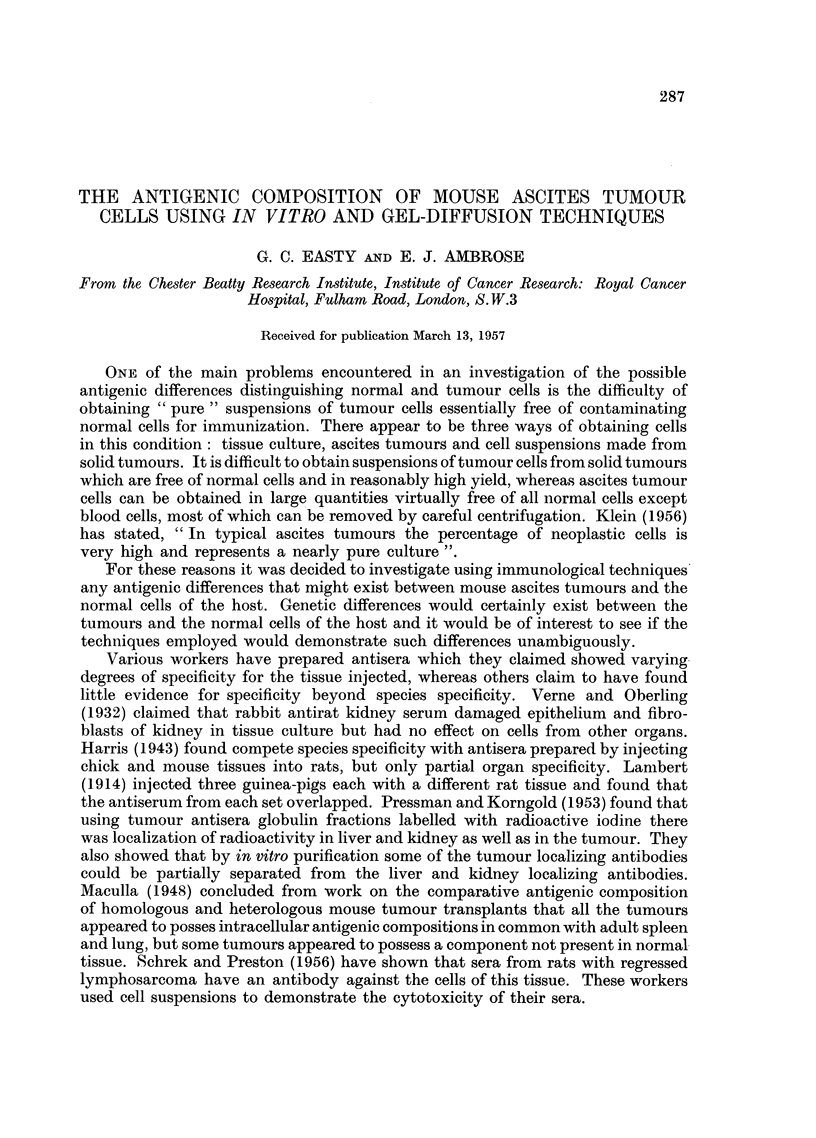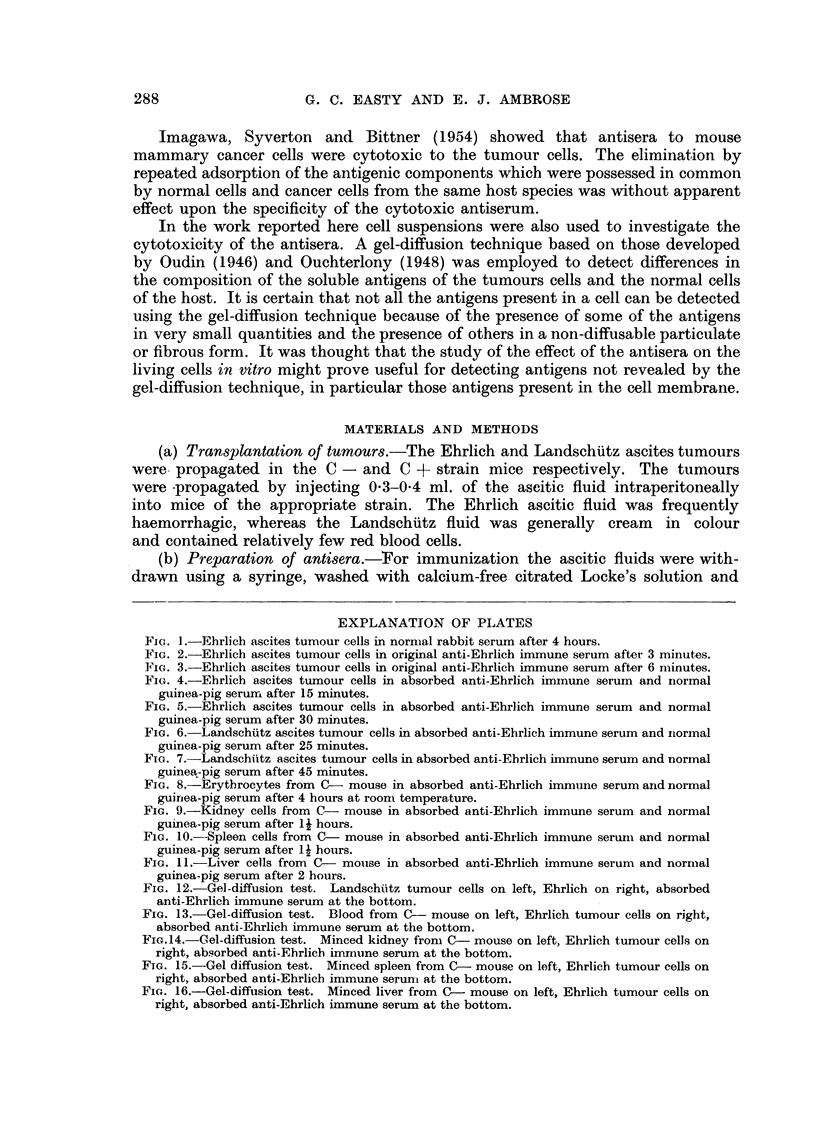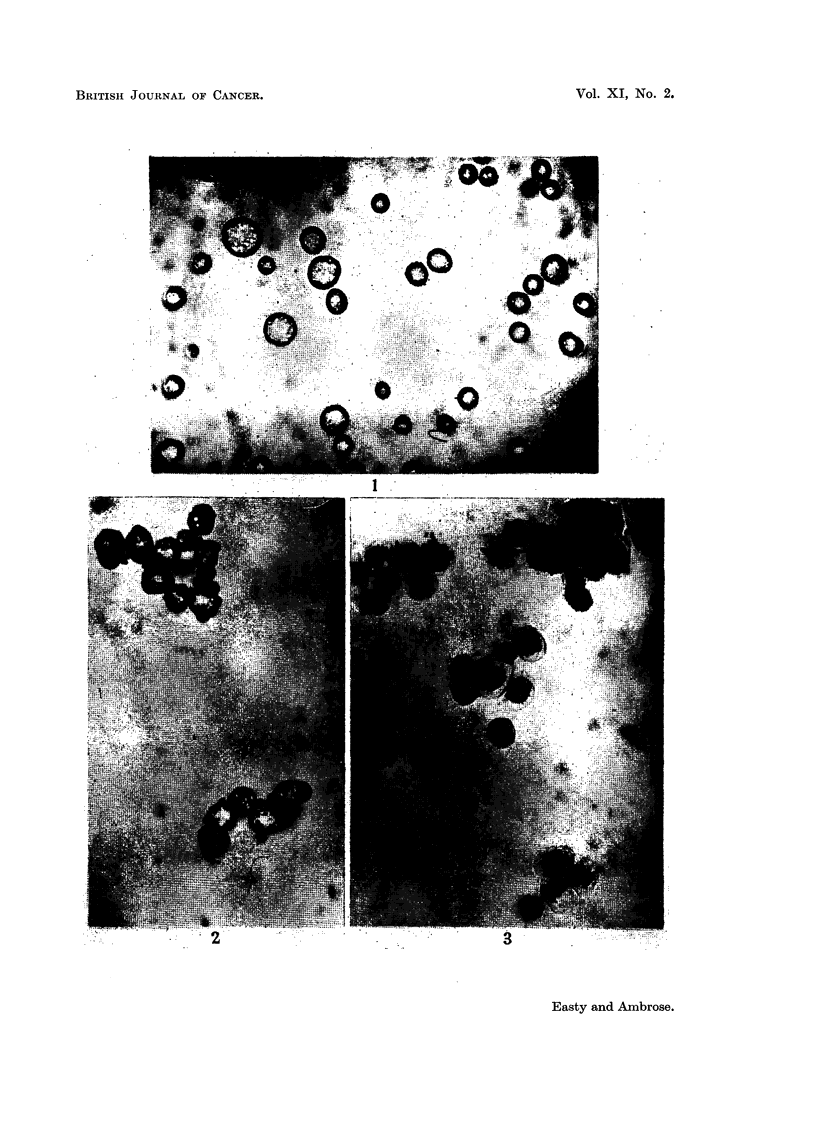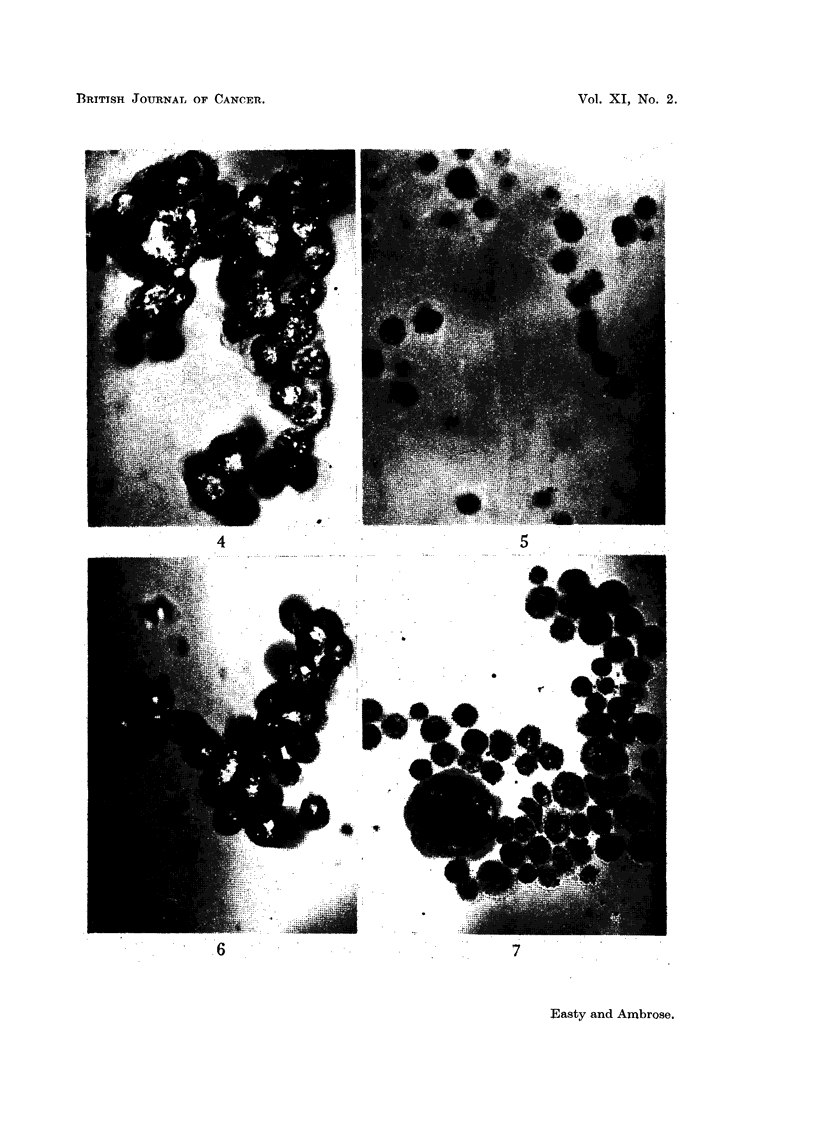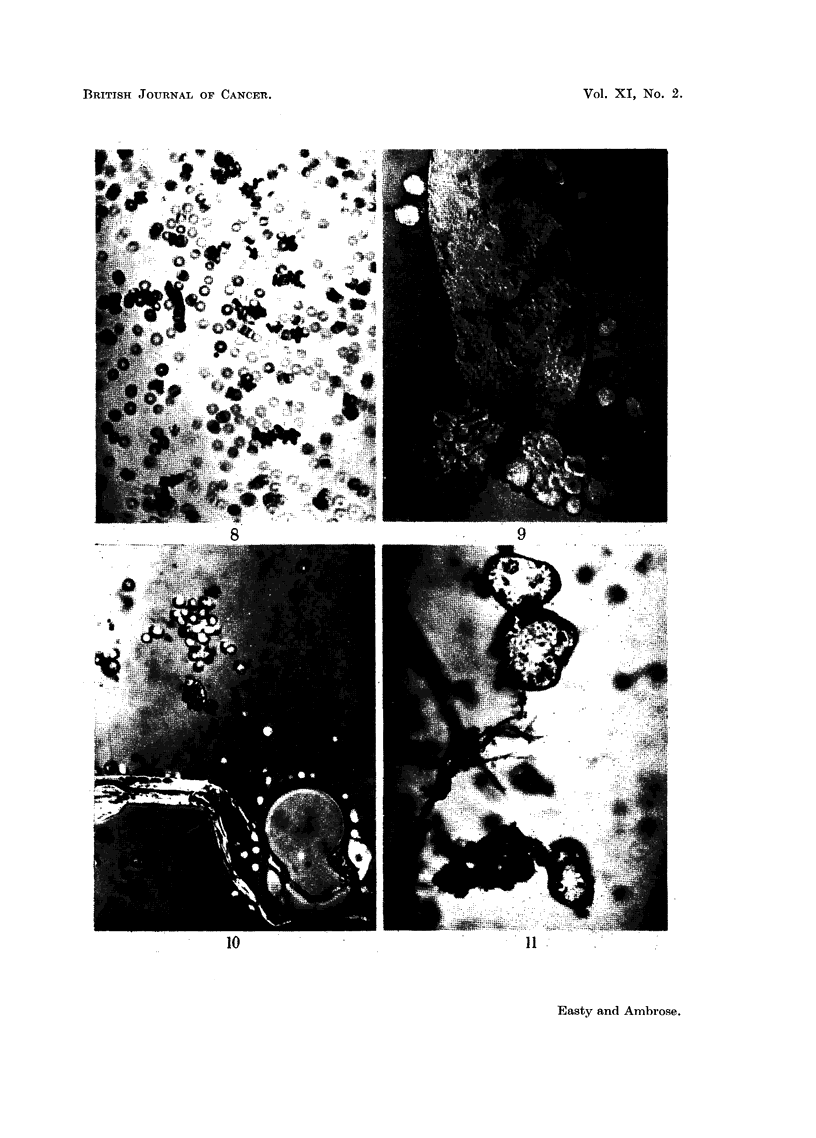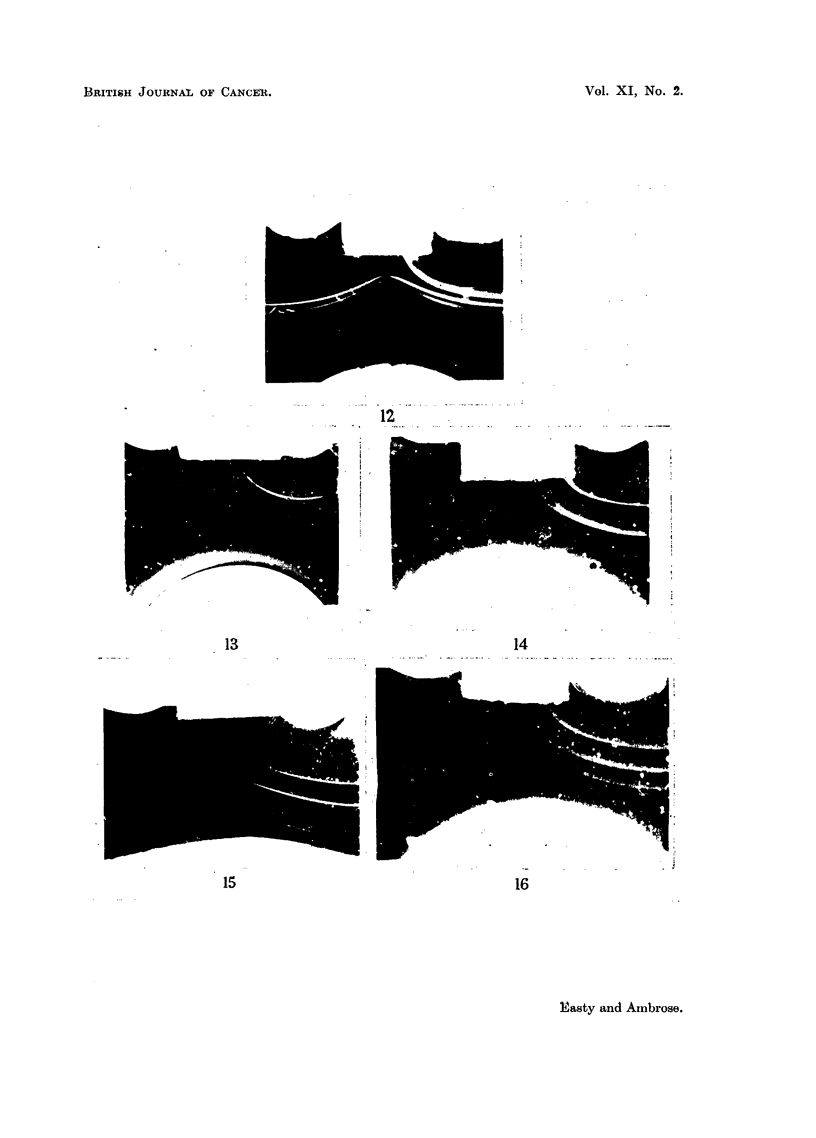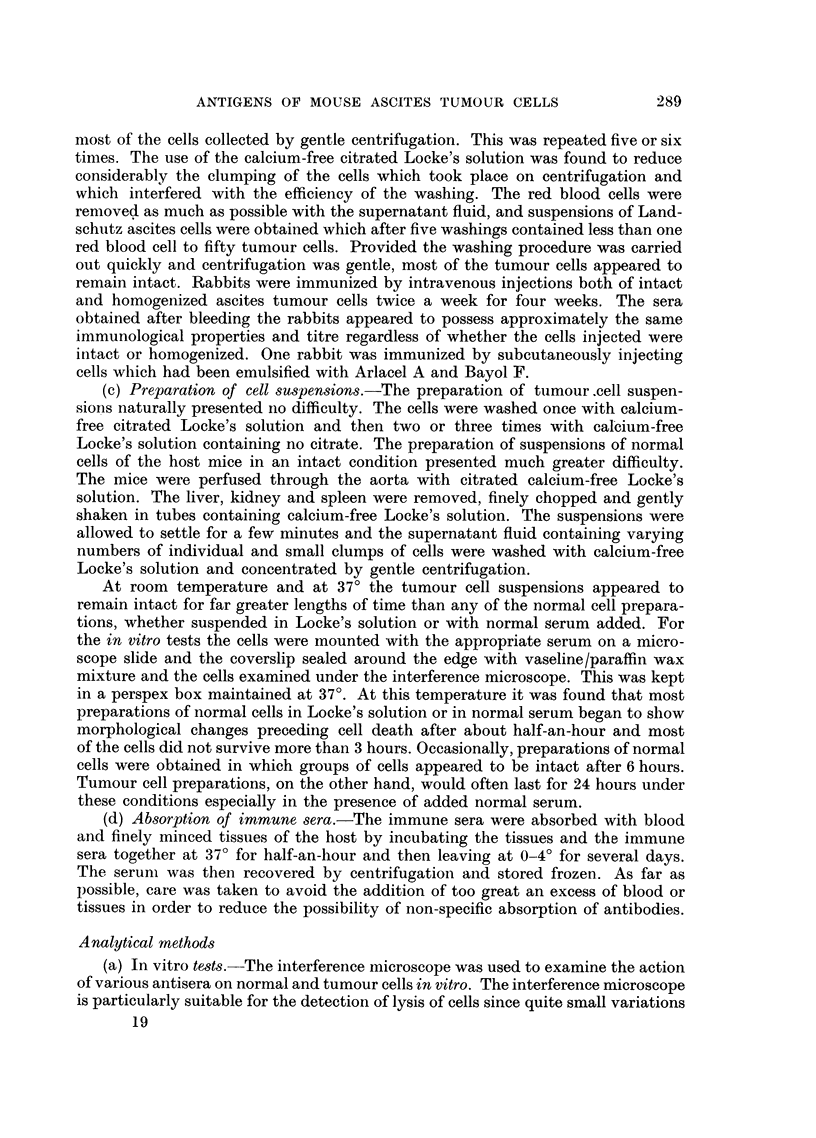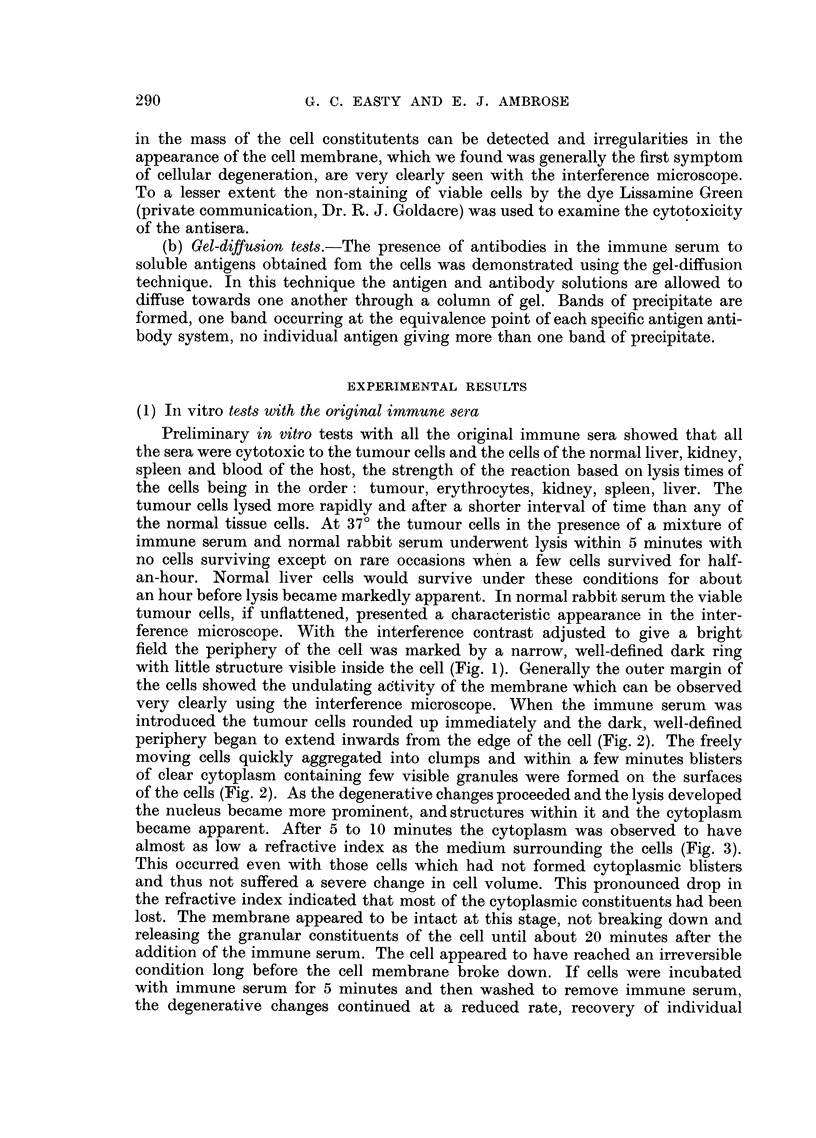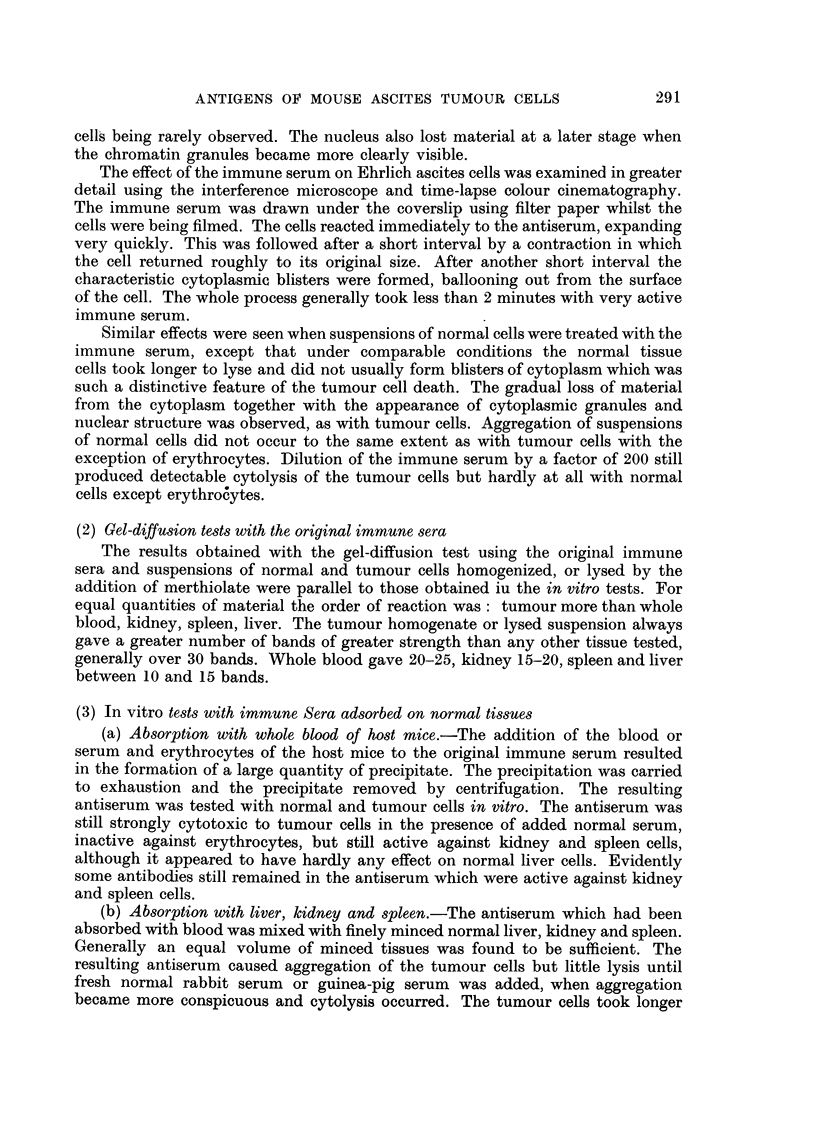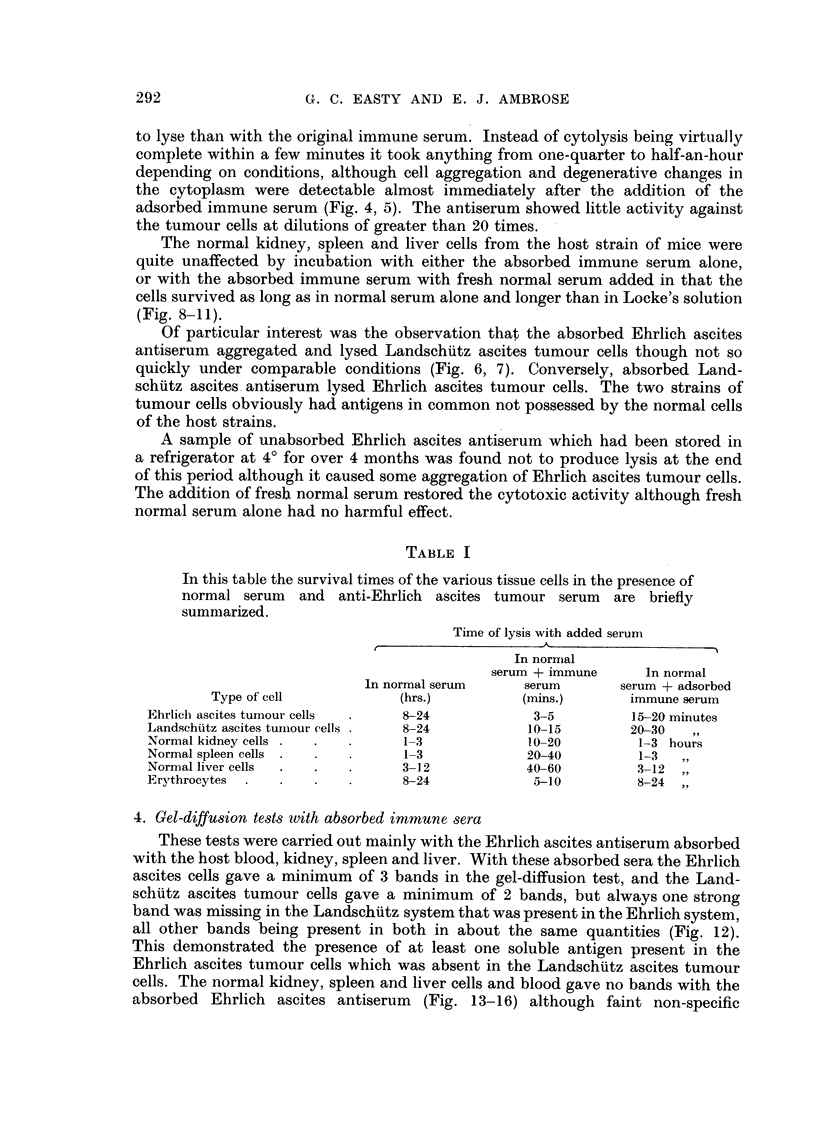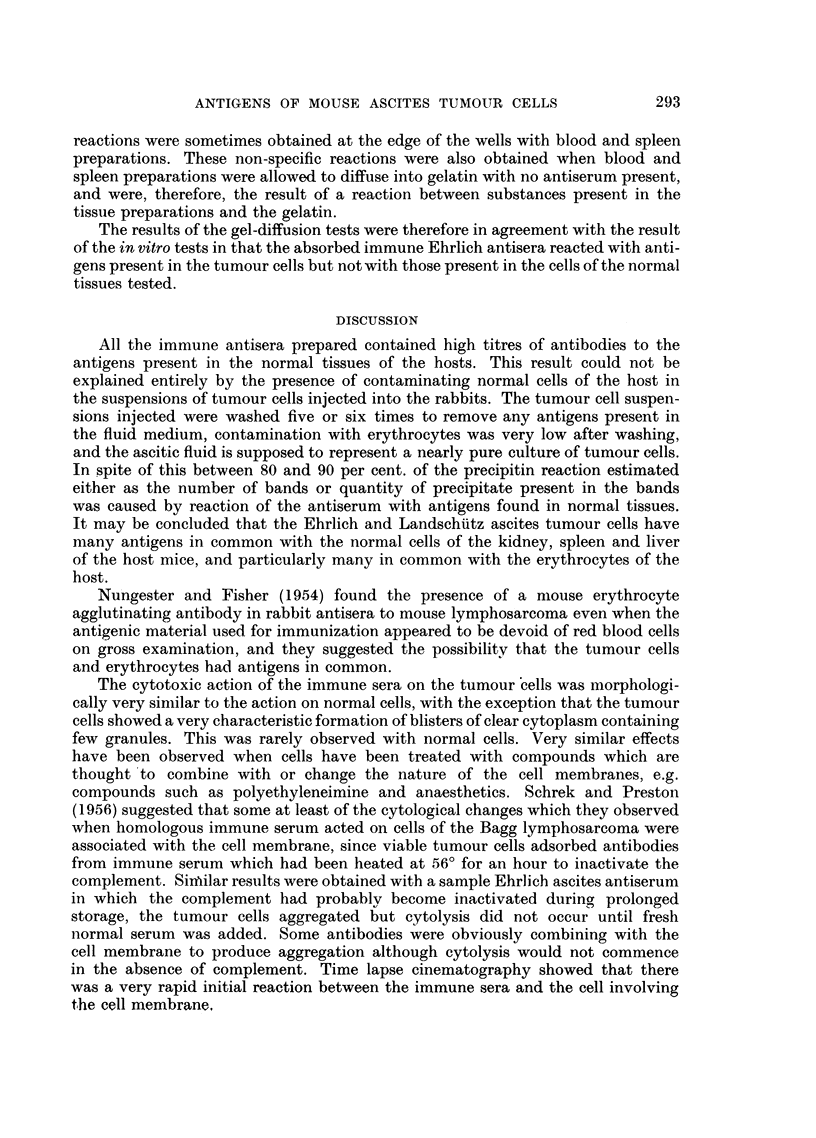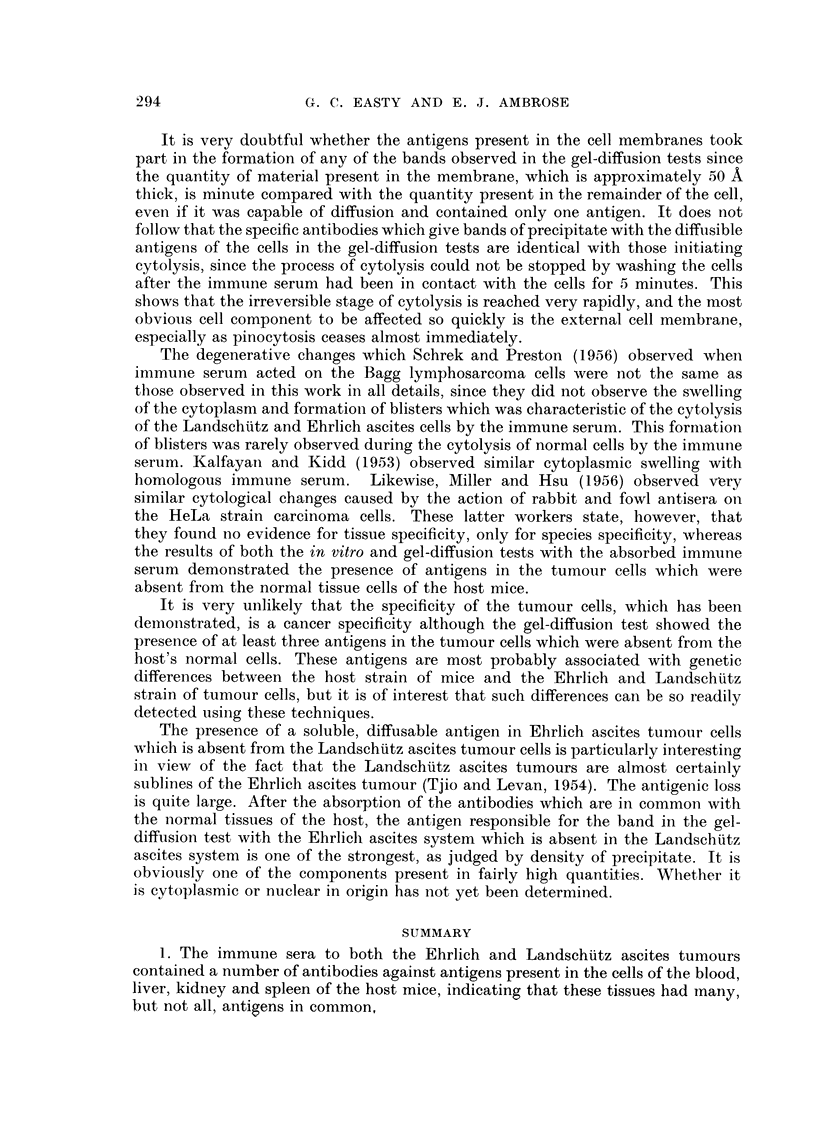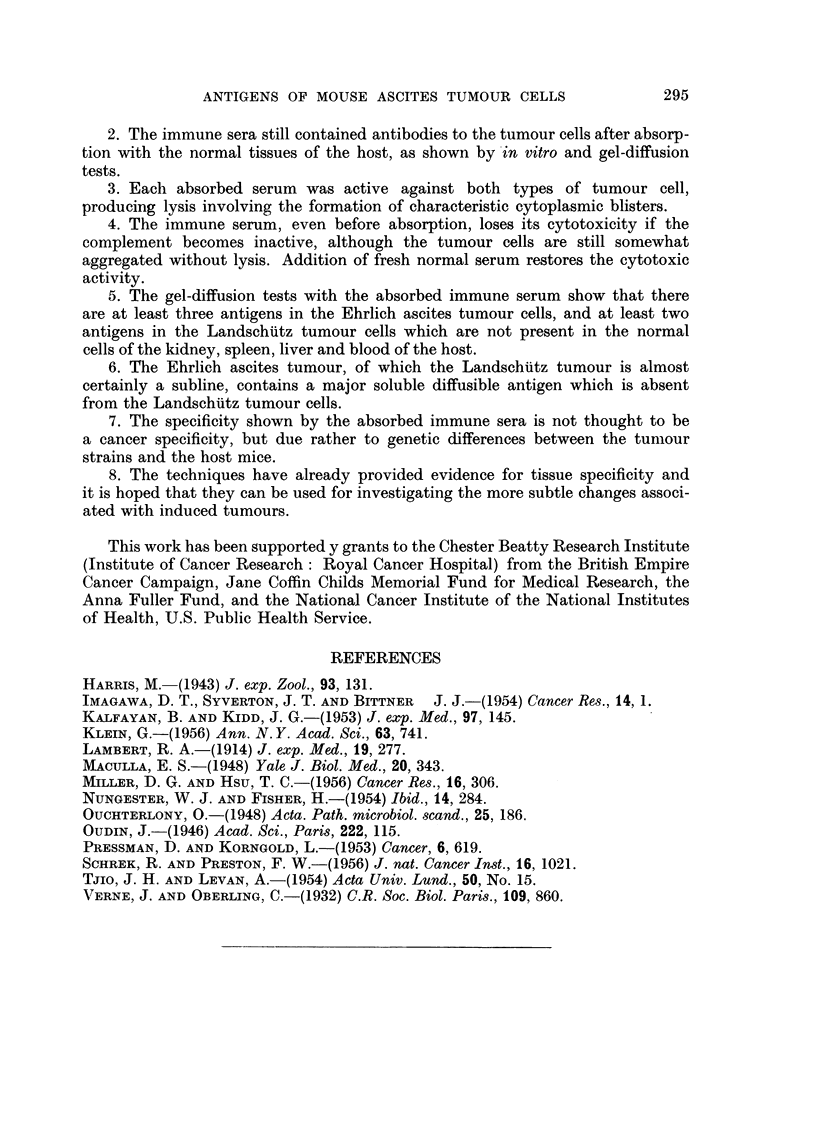# The Antigenic Composition of Mouse Ascites Tumour Cells Using In Vitro and Gel-diffusion Techniques

**DOI:** 10.1038/bjc.1957.37

**Published:** 1957-06

**Authors:** G. C. Easty, E. J. Ambrose

## Abstract

**Images:**


### THE ANTIGENIC COMPOSITION OF MOUSE ASCITES TUMOUR CELLS USING *IN VITRO* AND GEL-DIFFUSION TECHNIQUES

G. C. EASTY AND E. J. AMBROSE

*From the Chester Beatty Research Institute, Institute of Cancer Research: Royal Cancer Hospital, Fulham Road, London, S.W.3*

Received for publication March 13, 1957

ONE of the main problems encountered in an investigation of the possible antigenic differences distinguishing normal and tumour cells is the difficulty of obtaining " pure " suspensions of tumour cells essentially free of contaminating normal cells for immunization. There appear to be three ways of obtaining cells in this condition : tissue culture, ascites tumours and cell suspensions made from solid tumours. It is difficult to obtain suspensions of tumour cells from solid tumours which are free of normal cells and in reasonably high yield, whereas ascites tumour cells can be obtained in large quantities virtually free of all normal cells except blood cells, most of which can be removed by careful centrifugation. Klein (1956) has stated, " In typical ascites tumours the percentage of neoplastic cells is very high and represents a nearly pure culture ".

For these reasons it was decided to investigate using immunological techniques any antigenic differences that might exist between mouse ascites tumours and the normal cells of the host. Genetic differences would certainly exist between the tumours and the normal cells of the host and it would be of interest to see if the techniques employed would demonstrate such differences unambiguously.

Various workers have prepared antisera which they claimed showed varying degrees of specificity for the tissue injected, whereas others claim to have found little evidence for specificity beyond species specificity. Verne and Oberling (1932) claimed that rabbit antirat kidney serum damaged epithelium and fibroblasts of kidney in tissue culture but had no effect on cells from other organs. Harris (1943) found compete species specificity with antisera prepared by injecting chick and mouse tissues into rats, but only partial organ specificity. Lambert (1914) injected three guinea-pigs each with a different rat tissue and found that the antiserum from each set overlapped. Pressman and Korngold (1953) found that using tumour antisera globulin fractions labelled with radioactive iodine there was localization of radioactivity in liver and kidney as well as in the tumour. They also showed that by *in vitro* purification some of the tumour localizing antibodies could be partially separated from the liver and kidney localizing antibodies. Maculla (1948) concluded from work on the comparative antigenic composition of homologous and heterologous mouse tumour transplants that all the tumours appeared to posses intracellular antigenic compositions in common with adult spleen and lung, but some tumours appeared to possess a component not present in normal tissue. Schrek and Preston (1956) have shown that sera from rats with regressed lymphosarcoma have an antibody against the cells of this tissue. These workers used cell suspensions to demonstrate the cytotoxicity of their sera.

Imagawa, Syverton and Bittner (1954) showed that antisera to mouse mammary cancer cells were cytotoxic to the tumour cells. The elimination by repeated adsorption of the antigenic components which were possessed in common by normal cells and cancer cells from the same host species was without apparent effect upon the specificity of the cytotoxic antiserum.

In the work reported here cell suspensions were also used to investigate the cytotoxicity of the antisera. A gel-diffusion technique based on those developed by Oudin (1946) and Ouchterlony (1948) was employed to detect differences in the composition of the soluble antigens of the tumours cells and the normal cells of the host. It is certain that not all the antigens present in a cell can be detected using the gel-diffusion technique because of the presence of some of the antigens in very small quantities and the presence of others in a non-diffusable particulate or fibrous form. It was thought that the study of the effect of the antisera on the living cells *in vitro* might prove useful for detecting antigens not revealed by the gel-diffusion technique, in particular those antigens present in the cell membrane.

### MATERIALS AND METHODS

(a) *Transplantation of tumours.*—The Ehrlich and Landschütz ascites tumours were propagated in the C — and C + strain mice respectively. The tumours were propagated by injecting 0·3–0·4 ml. of the ascitic fluid intraperitoneally into mice of the appropriate strain. The Ehrlich ascitic fluid was frequently haemorrhagic, whereas the Landschütz fluid was generally cream in colour and contained relatively few red blood cells.

(b) *Preparation of antisera.*—For immunization the ascitic fluids were withdrawn using a syringe, washed with calcium-free citrated Locke's solution and

---

### EXPLANATION OF PLATES

FIG. 1.—Ehrlich ascites tumour cells in normal rabbit serum after 4 hours.
FIG. 2.—Ehrlich ascites tumour cells in original anti-Ehrlich immune serum after 3 minutes.
FIG. 3.—Ehrlich ascites tumour cells in original anti-Ehrlich immune serum after 6 minutes.
FIG. 4.—Ehrlich ascites tumour cells in absorbed anti-Ehrlich immune serum and normal guinea-pig serum after 15 minutes.
FIG. 5.—Ehrlich ascites tumour cells in absorbed anti-Ehrlich immune serum and normal guinea-pig serum after 30 minutes.
FIG. 6.—Landschütz ascites tumour cells in absorbed anti-Ehrlich immune serum and normal guinea-pig serum after 25 minutes.
FIG. 7.—Landschütz ascites tumour cells in absorbed anti-Ehrlich immune serum and normal guinea-pig serum after 45 minutes.
FIG. 8.—Erythrocytes from C— mouse in absorbed anti-Ehrlich immune serum and normal guinea-pig serum after 4 hours at room temperature.
FIG. 9.—Kidney cells from C— mouse in absorbed anti-Ehrlich immune serum and normal guinea-pig serum after 1½ hours.
FIG. 10.—Spleen cells from C— mouse in absorbed anti-Ehrlich immune serum and normal guinea-pig serum after 1½ hours.
FIG. 11.—Liver cells from C— mouse in absorbed anti-Ehrlich immune serum and normal guinea-pig serum after 2 hours.
FIG. 12.—Gel-diffusion test. Landschütz tumour cells on left, Ehrlich on right, absorbed anti-Ehrlich immune serum at the bottom.
FIG. 13.—Gel-diffusion test. Blood from C— mouse on left, Ehrlich tumour cells on right, absorbed anti-Ehrlich immune serum at the bottom.
FIG. 14.—Gel-diffusion test. Minced kidney from C— mouse on left, Ehrlich tumour cells on right, absorbed anti-Ehrlich immune serum at the bottom.
FIG. 15.—Gel diffusion test. Minced spleen from C— mouse on left, Ehrlich tumour cells on right, absorbed anti-Ehrlich immune serum at the bottom.
FIG. 16.—Gel-diffusion test. Minced liver from C— mouse on left, Ehrlich tumour cells on right, absorbed anti-Ehrlich immune serum at the bottom.

1

2

3

Easty and Ambrose.

4

5

6

7

Easty and Ambrose.

8

9

10

11

Easty and Ambrose.

12

13

14

15

16

Easty and Ambrose.

most of the cells collected by gentle centrifugation. This was repeated five or six times. The use of the calcium-free citrated Locke's solution was found to reduce considerably the clumping of the cells which took place on centrifugation and which interfered with the efficiency of the washing. The red blood cells were removed as much as possible with the supernatant fluid, and suspensions of Landschutz ascites cells were obtained which after five washings contained less than one red blood cell to fifty tumour cells. Provided the washing procedure was carried out quickly and centrifugation was gentle, most of the tumour cells appeared to remain intact. Rabbits were immunized by intravenous injections both of intact and homogenized ascites tumour cells twice a week for four weeks. The sera obtained after bleeding the rabbits appeared to possess approximately the same immunological properties and titre regardless of whether the cells injected were intact or homogenized. One rabbit was immunized by subcutaneously injecting cells which had been emulsified with Arlacel A and Bayol F.

(c) *Preparation of cell suspensions.*—The preparation of tumour cell suspensions naturally presented no difficulty. The cells were washed once with calcium-free citrated Locke's solution and then two or three times with calcium-free Locke's solution containing no citrate. The preparation of suspensions of normal cells of the host mice in an intact condition presented much greater difficulty. The mice were perfused through the aorta with citrated calcium-free Locke's solution. The liver, kidney and spleen were removed, finely chopped and gently shaken in tubes containing calcium-free Locke's solution. The suspensions were allowed to settle for a few minutes and the supernatant fluid containing varying numbers of individual and small clumps of cells were washed with calcium-free Locke's solution and concentrated by gentle centrifugation.

At room temperature and at 37° the tumour cell suspensions appeared to remain intact for far greater lengths of time than any of the normal cell preparations, whether suspended in Locke's solution or with normal serum added. For the *in vitro* tests the cells were mounted with the appropriate serum on a microscope slide and the coverslip sealed around the edge with vaseline/paraffin wax mixture and the cells examined under the interference microscope. This was kept in a perspex box maintained at 37°. At this temperature it was found that most preparations of normal cells in Locke's solution or in normal serum began to show morphological changes preceding cell death after about half-an-hour and most of the cells did not survive more than 3 hours. Occasionally, preparations of normal cells were obtained in which groups of cells appeared to be intact after 6 hours. Tumour cell preparations, on the other hand, would often last for 24 hours under these conditions especially in the presence of added normal serum.

(d) *Absorption of immune sera.*—The immune sera were absorbed with blood and finely minced tissues of the host by incubating the tissues and the immune sera together at 37° for half-an-hour and then leaving at 0–4° for several days. The serum was then recovered by centrifugation and stored frozen. As far as possible, care was taken to avoid the addition of too great an excess of blood or tissues in order to reduce the possibility of non-specific absorption of antibodies.

*Analytical methods*

(a) In vitro *tests.*—The interference microscope was used to examine the action of various antisera on normal and tumour cells *in vitro*. The interference microscope is particularly suitable for the detection of lysis of cells since quite small variations in the mass of the cell constitutents can be detected and irregularities in the appearance of the cell membrane, which we found was generally the first symptom of cellular degeneration, are very clearly seen with the interference microscope. To a lesser extent the non-staining of viable cells by the dye Lissamine Green (private communication, Dr. R. J. Goldacre) was used to examine the cytotoxicity of the antisera.

(b) *Gel-diffusion tests.*—The presence of antibodies in the immune serum to soluble antigens obtained fom the cells was demonstrated using the gel-diffusion technique. In this technique the antigen and antibody solutions are allowed to diffuse towards one another through a column of gel. Bands of precipitate are formed, one band occurring at the equivalence point of each specific antigen antibody system, no individual antigen giving more than one band of precipitate.

### EXPERIMENTAL RESULTS

### (1) In vitro *tests with the original immune sera*

Preliminary *in vitro* tests with all the original immune sera showed that all the sera were cytotoxic to the tumour cells and the cells of the normal liver, kidney, spleen and blood of the host, the strength of the reaction based on lysis times of the cells being in the order : tumour, erythrocytes, kidney, spleen, liver. The tumour cells lysed more rapidly and after a shorter interval of time than any of the normal tissue cells. At 37° the tumour cells in the presence of a mixture of immune serum and normal rabbit serum underwent lysis within 5 minutes with no cells surviving except on rare occasions when a few cells survived for half-an-hour. Normal liver cells would survive under these conditions for about an hour before lysis became markedly apparent. In normal rabbit serum the viable tumour cells, if unflattened, presented a characteristic appearance in the interference microscope. With the interference contrast adjusted to give a bright field the periphery of the cell was marked by a narrow, well-defined dark ring with little structure visible inside the cell (Fig. 1). Generally the outer margin of the cells showed the undulating activity of the membrane which can be observed very clearly using the interference microscope. When the immune serum was introduced the tumour cells rounded up immediately and the dark, well-defined periphery began to extend inwards from the edge of the cell (Fig. 2). The freely moving cells quickly aggregated into clumps and within a few minutes blisters of clear cytoplasm containing few visible granules were formed on the surfaces of the cells (Fig. 2). As the degenerative changes proceeded and the lysis developed the nucleus became more prominent, and structures within it and the cytoplasm became apparent. After 5 to 10 minutes the cytoplasm was observed to have almost as low a refractive index as the medium surrounding the cells (Fig. 3). This occurred even with those cells which had not formed cytoplasmic blisters and thus not suffered a severe change in cell volume. This pronounced drop in the refractive index indicated that most of the cytoplasmic constituents had been lost. The membrane appeared to be intact at this stage, not breaking down and releasing the granular constituents of the cell until about 20 minutes after the addition of the immune serum. The cell appeared to have reached an irreversible condition long before the cell membrane broke down. If cells were incubated with immune serum for 5 minutes and then washed to remove immune serum, the degenerative changes continued at a reduced rate, recovery of individual cells being rarely observed. The nucleus also lost material at a later stage when the chromatin granules became more clearly visible.

The effect of the immune serum on Ehrlich ascites cells was examined in greater detail using the interference microscope and time-lapse colour cinematography. The immune serum was drawn under the coverslip using filter paper whilst the cells were being filmed. The cells reacted immediately to the antiserum, expanding very quickly. This was followed after a short interval by a contraction in which the cell returned roughly to its original size. After another short interval the characteristic cytoplasmic blisters were formed, ballooning out from the surface of the cell. The whole process generally took less than 2 minutes with very active immune serum.

Similar effects were seen when suspensions of normal cells were treated with the immune serum, except that under comparable conditions the normal tissue cells took longer to lyse and did not usually form blisters of cytoplasm which was such a distinctive feature of the tumour cell death. The gradual loss of material from the cytoplasm together with the appearance of cytoplasmic granules and nuclear structure was observed, as with tumour cells. Aggregation of suspensions of normal cells did not occur to the same extent as with tumour cells with the exception of erythrocytes. Dilution of the immune serum by a factor of 200 still produced detectable cytolysis of the tumour cells but hardly at all with normal cells except erythrocytes.

(2) *Gel-diffusion tests with the original immune sera*

The results obtained with the gel-diffusion test using the original immune sera and suspensions of normal and tumour cells homogenized, or lysed by the addition of merthiolate were parallel to those obtained iu the *in vitro* tests. For equal quantities of material the order of reaction was : tumour more than whole blood, kidney, spleen, liver. The tumour homogenate or lysed suspension always gave a greater number of bands of greater strength than any other tissue tested, generally over 30 bands. Whole blood gave 20–25, kidney 15–20, spleen and liver between 10 and 15 bands.

(3) In vitro *tests with immune Sera adsorbed on normal tissues*

(a) *Absorption with whole blood of host mice.*—The addition of the blood or serum and erythrocytes of the host mice to the original immune serum resulted in the formation of a large quantity of precipitate. The precipitation was carried to exhaustion and the precipitate removed by centrifugation. The resulting antiserum was tested with normal and tumour cells *in vitro*. The antiserum was still strongly cytotoxic to tumour cells in the presence of added normal serum, inactive against erythrocytes, but still active against kidney and spleen cells, although it appeared to have hardly any effect on normal liver cells. Evidently some antibodies still remained in the antiserum which were active against kidney and spleen cells.

(b) *Absorption with liver, kidney and spleen.*—The antiserum which had been absorbed with blood was mixed with finely minced normal liver, kidney and spleen. Generally an equal volume of minced tissues was found to be sufficient. The resulting antiserum caused aggregation of the tumour cells but little lysis until fresh normal rabbit serum or guinea-pig serum was added, when aggregation became more conspicuous and cytolysis occurred. The tumour cells took longer to lyse than with the original immune serum. Instead of cytolysis being virtually complete within a few minutes it took anything from one-quarter to half-an-hour depending on conditions, although cell aggregation and degenerative changes in the cytoplasm were detectable almost immediately after the addition of the adsorbed immune serum (Fig. 4, 5). The antiserum showed little activity against the tumour cells at dilutions of greater than 20 times.

The normal kidney, spleen and liver cells from the host strain of mice were quite unaffected by incubation with either the absorbed immune serum alone, or with the absorbed immune serum with fresh normal serum added in that the cells survived as long as in normal serum alone and longer than in Locke's solution (Fig. 8–11).

Of particular interest was the observation that the absorbed Ehrlich ascites antiserum aggregated and lysed Landschütz ascites tumour cells though not so quickly under comparable conditions (Fig. 6, 7). Conversely, absorbed Landschütz ascites antiserum lysed Ehrlich ascites tumour cells. The two strains of tumour cells obviously had antigens in common not possessed by the normal cells of the host strains.

A sample of unabsorbed Ehrlich ascites antiserum which had been stored in a refrigerator at 4° for over 4 months was found not to produce lysis at the end of this period although it caused some aggregation of Ehrlich ascites tumour cells. The addition of fresh normal serum restored the cytotoxic activity although fresh normal serum alone had no harmful effect.

TABLE I

In this table the survival times of the various tissue cells in the presence of normal serum and anti-Ehrlich ascites tumour serum are briefly summarized.

| | Time of lysis with added serum | | |
|---|---|---|---|
| Type of cell | In normal serum (hrs.) | In normal serum + immune serum (mins.) | In normal serum + adsorbed immune serum |
| Ehrlich ascites tumour cells | 8–24 | 3–5 | 15–20 minutes |
| Landschütz ascites tumour cells | 8–24 | 10–15 | 20–30 ,, |
| Normal kidney cells | 1–3 | 10–20 | 1–3 hours |
| Normal spleen cells | 1–3 | 20–40 | 1–3 ,, |
| Normal liver cells | 3–12 | 40–60 | 3–12 ,, |
| Erythrocytes | 8–24 | 5–10 | 8–24 ,, |

4. *Gel-diffusion tests with absorbed immune sera*

These tests were carried out mainly with the Ehrlich ascites antiserum absorbed with the host blood, kidney, spleen and liver. With these absorbed sera the Ehrlich ascites cells gave a minimum of 3 bands in the gel-diffusion test, and the Landschütz ascites tumour cells gave a minimum of 2 bands, but always one strong band was missing in the Landschütz system that was present in the Ehrlich system, all other bands being present in both in about the same quantities (Fig. 12). This demonstrated the presence of at least one soluble antigen present in the Ehrlich ascites tumour cells which was absent in the Landschütz ascites tumour cells. The normal kidney, spleen and liver cells and blood gave no bands with the absorbed Ehrlich ascites antiserum (Fig. 13–16) although faint non-specific reactions were sometimes obtained at the edge of the wells with blood and spleen preparations. These non-specific reactions were also obtained when blood and spleen preparations were allowed to diffuse into gelatin with no antiserum present, and were, therefore, the result of a reaction between substances present in the tissue preparations and the gelatin.

The results of the gel-diffusion tests were therefore in agreement with the result of the *in vitro* tests in that the absorbed immune Ehrlich antisera reacted with antigens present in the tumour cells but not with those present in the cells of the normal tissues tested.

### DISCUSSION

All the immune antisera prepared contained high titres of antibodies to the antigens present in the normal tissues of the hosts. This result could not be explained entirely by the presence of contaminating normal cells of the host in the suspensions of tumour cells injected into the rabbits. The tumour cell suspensions injected were washed five or six times to remove any antigens present in the fluid medium, contamination with erythrocytes was very low after washing, and the ascitic fluid is supposed to represent a nearly pure culture of tumour cells. In spite of this between 80 and 90 per cent. of the precipitin reaction estimated either as the number of bands or quantity of precipitate present in the bands was caused by reaction of the antiserum with antigens found in normal tissues. It may be concluded that the Ehrlich and Landschütz ascites tumour cells have many antigens in common with the normal cells of the kidney, spleen and liver of the host mice, and particularly many in common with the erythrocytes of the host.

Nungester and Fisher (1954) found the presence of a mouse erythrocyte agglutinating antibody in rabbit antisera to mouse lymphosarcoma even when the antigenic material used for immunization appeared to be devoid of red blood cells on gross examination, and they suggested the possibility that the tumour cells and erythrocytes had antigens in common.

The cytotoxic action of the immune sera on the tumour cells was morphologically very similar to the action on normal cells, with the exception that the tumour cells showed a very characteristic formation of blisters of clear cytoplasm containing few granules. This was rarely observed with normal cells. Very similar effects have been observed when cells have been treated with compounds which are thought to combine with or change the nature of the cell membranes, e.g. compounds such as polyethyleneimine and anaesthetics. Schrek and Preston (1956) suggested that some at least of the cytological changes which they observed when homologous immune serum acted on cells of the Bagg lymphosarcoma were associated with the cell membrane, since viable tumour cells adsorbed antibodies from immune serum which had been heated at 56° for an hour to inactivate the complement. Similar results were obtained with a sample Ehrlich ascites antiserum in which the complement had probably become inactivated during prolonged storage, the tumour cells aggregated but cytolysis did not occur until fresh normal serum was added. Some antibodies were obviously combining with the cell membrane to produce aggregation although cytolysis would not commence in the absence of complement. Time lapse cinematography showed that there was a very rapid initial reaction between the immune sera and the cell involving the cell membrane.

It is very doubtful whether the antigens present in the cell membranes took part in the formation of any of the bands observed in the gel-diffusion tests since the quantity of material present in the membrane, which is approximately 50 Å thick, is minute compared with the quantity present in the remainder of the cell, even if it was capable of diffusion and contained only one antigen. It does not follow that the specific antibodies which give bands of precipitate with the diffusible antigens of the cells in the gel-diffusion tests are identical with those initiating cytolysis, since the process of cytolysis could not be stopped by washing the cells after the immune serum had been in contact with the cells for 5 minutes. This shows that the irreversible stage of cytolysis is reached very rapidly, and the most obvious cell component to be affected so quickly is the external cell membrane, especially as pinocytosis ceases almost immediately.

The degenerative changes which Schrek and Preston (1956) observed when immune serum acted on the Bagg lymphosarcoma cells were not the same as those observed in this work in all details, since they did not observe the swelling of the cytoplasm and formation of blisters which was characteristic of the cytolysis of the Landschütz and Ehrlich ascites cells by the immune serum. This formation of blisters was rarely observed during the cytolysis of normal cells by the immune serum. Kalfayan and Kidd (1953) observed similar cytoplasmic swelling with homologous immune serum. Likewise, Miller and Hsu (1956) observed very similar cytological changes caused by the action of rabbit and fowl antisera on the HeLa strain carcinoma cells. These latter workers state, however, that they found no evidence for tissue specificity, only for species specificity, whereas the results of both the *in vitro* and gel-diffusion tests with the absorbed immune serum demonstrated the presence of antigens in the tumour cells which were absent from the normal tissue cells of the host mice.

It is very unlikely that the specificity of the tumour cells, which has been demonstrated, is a cancer specificity although the gel-diffusion test showed the presence of at least three antigens in the tumour cells which were absent from the host's normal cells. These antigens are most probably associated with genetic differences between the host strain of mice and the Ehrlich and Landschütz strain of tumour cells, but it is of interest that such differences can be so readily detected using these techniques.

The presence of a soluble, diffusable antigen in Ehrlich ascites tumour cells which is absent from the Landschütz ascites tumour cells is particularly interesting in view of the fact that the Landschütz ascites tumours are almost certainly sublines of the Ehrlich ascites tumour (Tjio and Levan, 1954). The antigenic loss is quite large. After the absorption of the antibodies which are in common with the normal tissues of the host, the antigen responsible for the band in the gel-diffusion test with the Ehrlich ascites system which is absent in the Landschütz ascites system is one of the strongest, as judged by density of precipitate. It is obviously one of the components present in fairly high quantities. Whether it is cytoplasmic or nuclear in origin has not yet been determined.

### SUMMARY

1. The immune sera to both the Ehrlich and Landschütz ascites tumours contained a number of antibodies against antigens present in the cells of the blood, liver, kidney and spleen of the host mice, indicating that these tissues had many, but not all, antigens in common.

2. The immune sera still contained antibodies to the tumour cells after absorption with the normal tissues of the host, as shown by *in vitro* and gel-diffusion tests.

3. Each absorbed serum was active against both types of tumour cell, producing lysis involving the formation of characteristic cytoplasmic blisters.

4. The immune serum, even before absorption, loses its cytotoxicity if the complement becomes inactive, although the tumour cells are still somewhat aggregated without lysis. Addition of fresh normal serum restores the cytotoxic activity.

5. The gel-diffusion tests with the absorbed immune serum show that there are at least three antigens in the Ehrlich ascites tumour cells, and at least two antigens in the Landschütz tumour cells which are not present in the normal cells of the kidney, spleen, liver and blood of the host.

6. The Ehrlich ascites tumour, of which the Landschütz tumour is almost certainly a subline, contains a major soluble diffusible antigen which is absent from the Landschütz tumour cells.

7. The specificity shown by the absorbed immune sera is not thought to be a cancer specificity, but due rather to genetic differences between the tumour strains and the host mice.

8. The techniques have already provided evidence for tissue specificity and it is hoped that they can be used for investigating the more subtle changes associated with induced tumours.

This work has been supported y grants to the Chester Beatty Research Institute (Institute of Cancer Research : Royal Cancer Hospital) from the British Empire Cancer Campaign, Jane Coffin Childs Memorial Fund for Medical Research, the Anna Fuller Fund, and the National Cancer Institute of the National Institutes of Health, U.S. Public Health Service.

### REFERENCES

Harris, M.—(1943) *J. exp. Zool.*, **93**, 131.
Imagawa, D. T., Syverton, J. T. and Bittner J. J.—(1954) *Cancer Res.*, **14**, 1.
Kalfayan, B. and Kidd, J. G.—(1953) *J. exp. Med.*, **97**, 145.
Klein, G.—(1956) *Ann. N.Y. Acad. Sci.*, **63**, 741.
Lambert, R. A.—(1914) *J. exp. Med.*, **19**, 277.
Maculla, E. S.—(1948) *Yale J. Biol. Med.*, **20**, 343.
Miller, D. G. and Hsu, T. C.—(1956) *Cancer Res.*, **16**, 306.
Nungester, W. J. and Fisher, H.—(1954) *Ibid.*, **14**, 284.
Ouchterlony, O.—(1948) *Acta. Path. microbiol. scand.*, **25**, 186.
Oudin, J.—(1946) *Acad. Sci., Paris*, **222**, 115.
Pressman, D. and Korngold, L.—(1953) *Cancer*, **6**, 619.
Schrek, R. and Preston, F. W.—(1956) *J. nat. Cancer Inst.*, **16**, 1021.
Tjio, J. H. and Levan, A.—(1954) *Acta Univ. Lund.*, **50**, No. 15.
Verne, J. and Oberling, C.—(1932) *C.R. Soc. Biol. Paris.*, **109**, 860.